# The Common Ice Plant (*Mesembryanthemum crystallinum* L.)–Phytoremediation Potential for Cadmium and Chromate-Contaminated Soils

**DOI:** 10.3390/plants9091230

**Published:** 2020-09-18

**Authors:** Marta Śliwa-Cebula, Paweł Kaszycki, Adriana Kaczmarczyk, Michał Nosek, Agnieszka Lis-Krzyścin, Zbigniew Miszalski

**Affiliations:** 1 Department of Plant Biology and Biotechnology, Faculty of Biotechnology and Horticulture, University of Agriculture in Krakow, al. 29 Listopada 54, 31-425 Kraków, Poland; marta.sliwa-cebula@student.urk.edu.pl (M.Ś.-C.); agnieszka.lis-krzyscin@urk.edu.pl (A.L.-K.); 2 The Franciszek Górski Institute of Plant Physiology Polish Academy of Sciences, Niezapominajek 21, 30-239 Kraków, Poland; a.kapron@ifr-pan.edu.pl; 3 Institute of Biology, Pedagogical University, Podchorążych 2, 30-084 Kraków, Poland; michal.nosek@up.krakow.pl; 4 Malopolska Centre of Biotechnology, Jagiellonian University, Gronostajowa 7a, 30-387 Kraków, Poland

**Keywords:** plant stress tolerance, heavy metal stress, chromate, cadmium, phytoextraction, phytostabilization, hyperaccumulation, soil remediation

## Abstract

The common ice plant (*Mesembryanthemum crystallinum* L.) is a widely studied model due to its tolerance to numerous biotic and abiotic stresses. In this study, carried out in model pots, the plants were treated with variant doses of Cd(II) and Cr(VI) and proved resistant to extreme levels of these heavy metals. Initial toxicity symptoms were observed upon final concentrations of 818 mg Cd kg^−1^ soil d.w., and 1699 mg Cr kg^−1^ applied as potassium chromate. Biometric analyses revealed that none of the Cr(VI) doses affected dry weight of the plant organs thus maintaining the shoot-to-root ratio. The Cd and Cr hypertolerance strategies were divergent and resulted in different accumulation patterns. For the case of Cd(II), an excluder-like mechanism was developed to prevent the plant from toxicity. For chromate, high accumulation potential together with Cr(VI) root-to-shoot translocation at sublethal concentrations was revealed (up to 6152 mg Cr kg^−1^ shoot at 4248 mg Cr kg^−1^ soil). It is concluded that *M. crystallinum* reveals considerable phytoremediation capabilities due to unique growth potential in contaminated substrates and is suitable for bioreclamation of degraded soils. The plant is especially applicable for efficient phytoextraction of chromate-contamination, whereas for Cd-affected areas it may have a phytostabilizing effect.

## 1. Introduction

The environmental impact of progressing anthropogenic activity brings severe risk to the bio- and geosphere. Among the main factors contributing to land degradation and deterioration are rapid industrial development, poor spatial planning, excessive exploitation of resources together with imbalanced water and soil management related to improper agricultural use. According to recent estimates, degraded soils contribute up to 24% of the global land area (approx. 35 million km^2^) [1,2]. Soil degradation results from numerous processes that negatively influence critical properties of soil environment such as chemical, physical characteristics and biological activity. Among the dominant factors responsible for industrial soil degradation are chemical pollutants which include both recalcitrant organic compounds (xenobiotics) and inorganic contaminants. The latter group covers a very divergent list of substances, namely acidic and alkali chemicals, salts, biogenic elements causing eutrophication, and, in particular, heavy metals, metallo-organic and inorganic complexes and derivatives. 

High soil salinity and contamination with heavy metals are problems of particular environmental concern and often occur concomitantly as a result of industrial activities [3,4,5,6]. Main sources of contamination with heavy metals and metalloids are mining, metallurgy, transport, tanneries, paint and wood protection industries, production of plastics and mineral fertilizers. Although several heavy metals (e.g., Cu, Zn, Fe, Mn, Ni, Co) are necessary micro- or ultra-elements that enable proper functioning of many organisms, they become inhibitory or toxic towards soil microbiota, plants, animals and humans when occur at higher concentrations and in easy bioavailable or reactive forms [7]. Cadmium and chromium can serve as examples of particularly hazardous elements; they both appear in ground-water environments mostly anthropogenically and are considered as priority pollutants by the U.S. EPA [8]. Moreover, cadmium-containing compounds are considered as priority hazardous substances by the European Commission [9]. According to the recent regulations in Poland [10] referring to surface soil layers for use in agriculture, chromium and cadmium have been listed among the substances bearing particular risk in terms of protection of ground surface. Permissible limits for the total content of each metal have been established and their values, depending on the soil subgroup, range from 2 to 5 mg kg^−1^ soil d.w. for Cd, and from 150 to 500 mg kg^−1^ for Cr. 

Cadmium, together with several other cytotoxic heavy metals and metalloids like Pb, Hg or As, has no known physiological or biochemical functions [7]. Its high direct toxicity towards plants includes oxidative stress, genotoxicity, impaired respiration and photosynthetic apparatus malfunctions [11,12,13]. Moreover, Cd^2+^ is taken up by plants in a competitive manner by means of divalent cation transporters due to its chemical structure similarities with several biologically important macro- and micronutrients such as Zn, Ca or Mg. Then, Cd undergoes translocation from roots to aerial plant parts, although, in general, the process is kept at a low level. In consequence, its accumulation can lead to deficiency of plant-beneficial elements, which can further contribute to the detrimental effect [11,12,13,14,15]. 

Chromium exhibits very complex chemistry and it may occur in a broad range of oxidation states (+1 to +6) characterized by various reactivities and solubilities, which lead to different ecotoxicities and environmental hazards [16,17,18]. Among the variant valence states, Cr(III) and Cr(VI) (chromate) are the most common forms and the latter one is considered the most hazardous to living organisms since it is genotoxic and mutagenic, causes plant growth inhibition, oxidative stress, interferes with nutrient uptake, and negatively affects photosynthesis [19,20,21,22]. The chromate ion (CrO_4_^2−^) structurally resembles the sulfate anion (SO_4_^2−^) and therefore it is actively incorporated by plant cells through nonspecific anionic transporter systems, especially by sulfate carriers [23,24]. The mechanisms of chromate accumulation, metabolism and toxicity have become subjects of thorough studies for a number of terrestrial plants [19,21,25]. It was shown that upon Cr(VI) uptake, most plants tend to retain this metal within the root tissue, keeping its translocation into shoots at the relatively low level and thus protecting aerial assimilative organs against impairment.

Chromate contaminants are discharged by numerous industrial activities [26,27]. Upon release to the environment, they usually undergo reduction to trivalent species and tend to bind to organic matter in soils forming insoluble or colloidal Cr(III) particles in sediments of water reservoirs [28]. Trivalent chromium is considered the most stable form, relatively immobile and the least biologically available [16,17]. However, chromates can still persist in well oxygenated systems [28]. Moreover, under certain oxidizing conditions such as in Mn-rich soils [29,30], in waters or at water-sediment interfaces in the presence of Mn-oxides [31], Cr(VI) may re-occur and become remobilized upon oxidation of Cr(III).

In efforts aimed at reclamation of polluted soils and rehabilitation of degraded environments, phytotechnologies including phytoremediation [7,32,33,34] appear as promising approaches that enable elaboration of cheap, non-invasive and efficient industrial-scale methods aimed at environmental recovery [18,23,35]. For cases of contamination with heavy metals, various strategies based on biotechnologically robust plants may be considered [36,37]. Since heavy metals cannot become biodegraded, possible technologies should include the application of highly tolerant plants for phytostabilization of affected areas, cultivation of plants capable of rhizoremediation and pollutant biotransformation, or the use of (hyper) accumulators capable of enhanced uptake of metals and their translocation into shoots. It is thus of high importance to search for new and powerful plants with phytoremediation potential that might prove applicable in remediation actions.

The common ice plant (*Mesembryanthemum crystallinum* L.) is seemingly a good candidate for phytoremediation actions due to its unusual properties. It is a semi-halophytic, fast-growing plant of the Aizoaceae family, exhibiting low nutritional and environmental requirements for growth and producing edible leaves and seeds. It can adapt to extreme conditions and is able to develop resistance mechanisms against numerous environmental stresses [38,39,40,41,42]. For many years it has served as a laboratory model suitable for studies on plant stress physiology and photosynthetic metabolism, especially due to its peculiar characteristics as a C_3_ – CAM intermediate plant [43,44,45]. 

The present work has been inspired by earlier contributions that evidenced high *M. crystallinum* tolerance to NaCl while showing extreme saline accumulation capabilities [41] as well as efficient uptake of Ni [3], Cd [46], Cu and Zn ions [47]. The aim of this study was to bring novel information about the common ice plant resistance against cadmium and chromate ions, with a special emphasis put on Cr accumulation and translocation from roots to shoots. Although the plant’s tolerance towards either Cd(II) or Cr(VI) as well as its metal accumulation levels were examined under laboratory model conditions (pot tests), we believe that the presented research results can reveal *M. crystallinum* usability in terms of phytoremediation of contaminated soils in environmental bioreclamation projects. The aim of this study was to bring novel information about the common ice plant resistance against cadmium and chromate ions.

## 2. Results 

### 2.1. Evaluation of Cadmium and Chromate Toxicities

**Figure 1:**
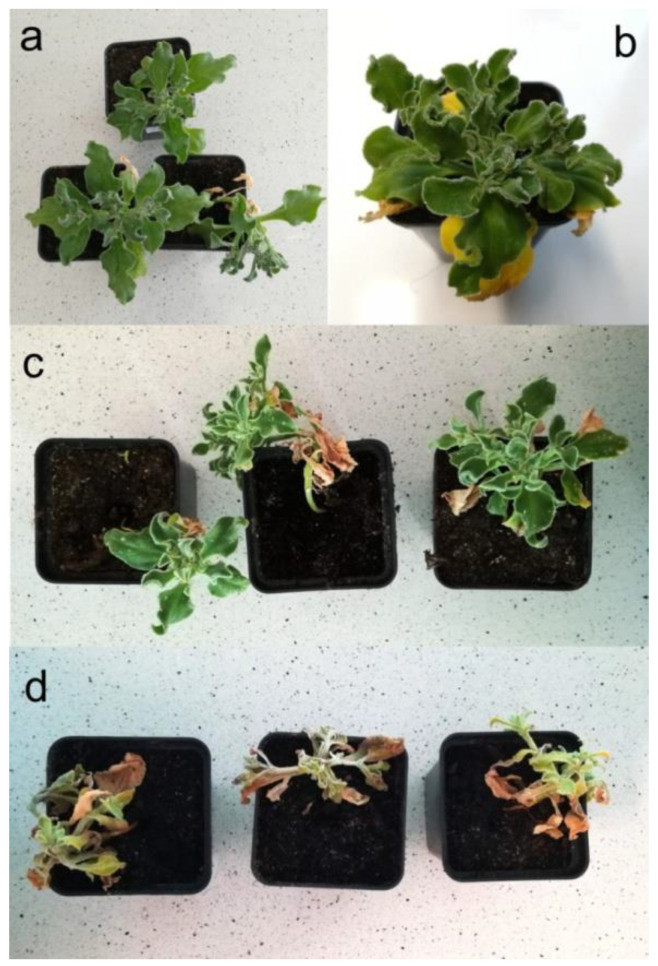
Morphological evaluation of toxicity symptoms caused by treatment of the common ice plant (*M. crystallinum*) with cadmium chloride (**b**) and potassium chromate (**c**,**d**). (**a**) control plants; (**b**) Cd treatment (800 µmol per pot = 818 mg kg^−1^ soil d.w., initial toxicity symptoms upon 8-day incubation); (**c**) chromate (3.6 mmol per pot = 1699 mg kg^−1^ soil d.w., initial toxicity symptoms upon 9-day incubation, concentration considered sub-lethal); (**d**) chromate (4.5 mmol per pot = 2124 mg kg^−1^ soil d.w., lethal concentration).

*Mesembryanthemum crystallinum* tolerance to the presence of the two tested heavy metals was first evaluated based on the morphological visible symptoms. For the case of Cd, the results were consistent with our earlier observations on high plant resistance to this metal [48,49]. Accordingly, no visible toxicity effects were developed up to the administered dose of 80 μmol Cd^2+^ per pot, that is 82 mg kg^−1^ soil d.w. (plants indistinguishable from the control ones, Figure 1a). Only at the highest Cd concentration (800 μmol per pot = 818 mg kg^−1^ soil d.w.) and after 8-day treatment, the first leaf pair turned yellow due to chlorosis, while the rest of the plant remained unaffected (Figure 1b). 

For chromate treatment, neither plant growth nor shoot and root morphologies were disturbed by the presence of Cr(VI) administered up to the final doses of 2.3 mmol Cr per pot (1086 mg Cr kg^−1^ soil d.w.). For the two treatment models tested, i.e., the use of either K_2_CrO_4_ or K_2_Cr_2_O_7_ as sources of Cr(VI) ions, diverse plant toxicity thresholds were observed. Upon irrigation with potassium chromate solution, after 9-day incubation, the leaves became partially necrotic with visible chlorosis symptoms at the final Cr concentration of 1699 mg kg^−1^ soil d.w. (3.6 mmol per pot; Figure 1c). When potassium dichromate was used, analogous manifestations were observed at the level of 3400 mg Cr kg^−1^ soil d.w. (7.2 mmol Cr(VI) per pot). At higher chromate levels, prolonged cultivation led to the final death of plants (Figure 1d). 

Biometric analyses of the common ice plant response to chromate treatment confirmed that Cr(VI) had no detrimental effects on soil-grown plants at doses up to 2.3 and 4.5 mmol per pot for irrigation with potassium chromate and dichromate, respectively (Table 1). Moreover, none of the applied concentrations led to significant modifications of the dry weight of roots and shoots, which in consequence maintained the dry weight of the whole plants and shoot-to-root (d.w.) ratio. Only the fresh weight analysis of the whole plants showed the total biomass (f.w.) decrease at high Cr(VI) levels, thus indicating alterations in the plant water status (Table 1).

### 2.2. Cd and Cr Accumulation Capacities

**Figure 2:**
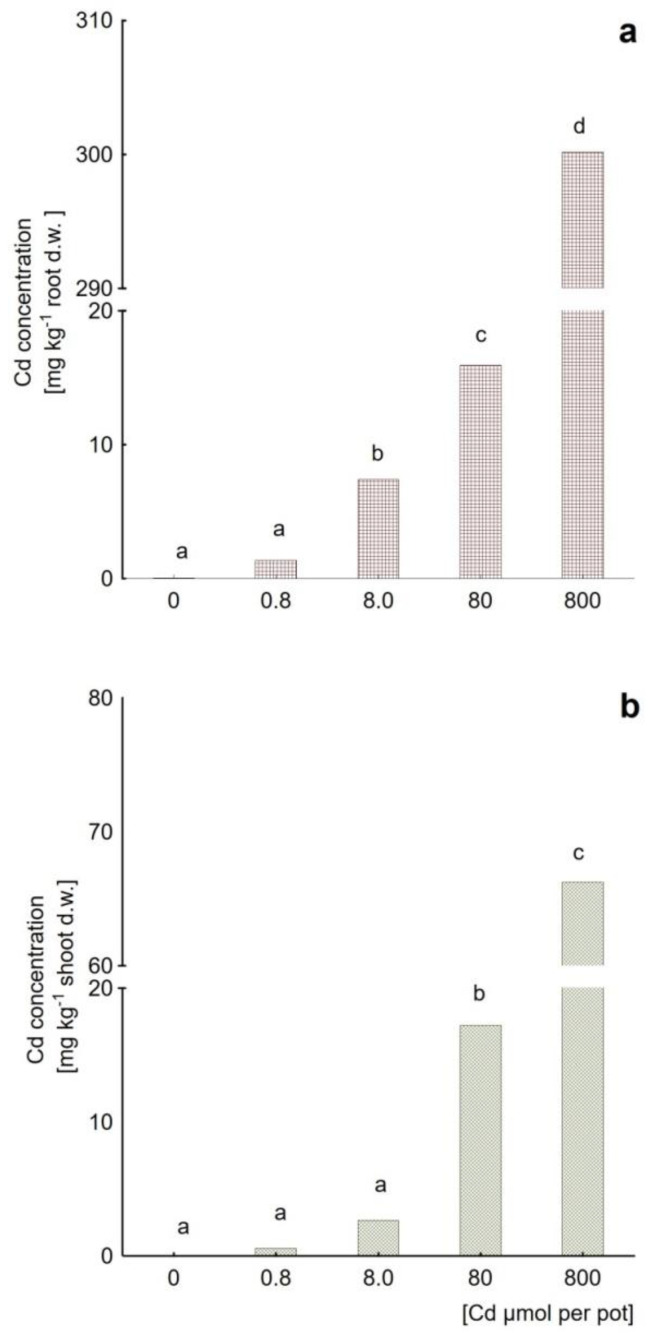
Cadmium content in (**a**) roots and (**b**) shoots of *Mesembryanthemum crystallinum* plants subjected to different Cd doses, ranging from 0.8 to 800 μmol of Cd applied per pot (untreated control at dose 0). Different letters above the bars indicate statistically significant differences at *p* ≤ 0.05.

**Figure 3:**
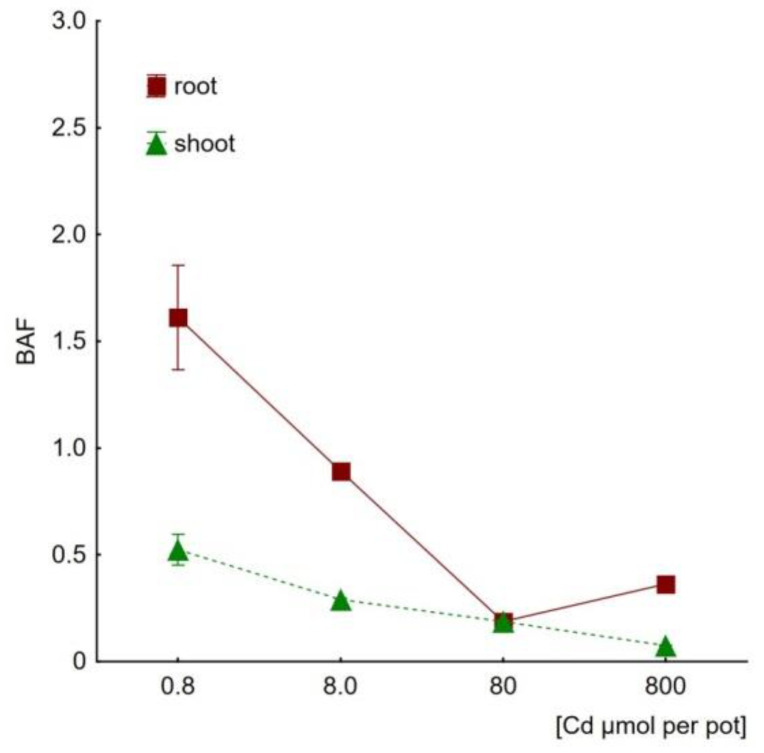
Cadmium bioaccumulation factors (BAF) of *Mesembryanthemum crystallinum* roots and shoots as determined for plants subjected to treatment with different Cd doses, ranging from 0.8 to 800 μmol of Cd applied per pot (N = 3, mean values ±SD).

The plants treated with both metal salts were tested for their accumulation capabilities of roots and the aerial parts (shoots). The results suggest different strategies of coping with the metals presence and their toxicity. Figure 2 shows cadmium accumulation capacities determined for all the variant Cd doses. The highest Cd levels were detected at a total applied dose of 0.8 mmol per pot (818 mg kg^−1^ soil d.w.) and was equal to 297 ± 8.30 and 61.09 ± 1.62 mg kg^−1^ of roots and shoots, respectively. Although the levels of Cd tended to grow in both roots (Figure 2a) and shoots (Figure 2b) along with the metal amount administered during irrigation, a more detailed analysis suggests the involvement of the excluder’s strategy. For this purpose, bioaccumulation (BAF) and translocation (TF) factors were considered. The BAF parameter reflects the efficiency of metal uptake from soil, whereas TF indicates the tendency to transport of a given pollutant from roots to shoots. The calculated BAF values (Figure 3) imply hindered Cd uptake by roots (BAF decreased with the increasing Cd doses) as well as blocked translocation to leaves (the decreasing BAF values reached as low as 0.07 ± 0.01 for the highest dose). These observations are supported by TF values <1.0 for all the applied Cd doses with the lowest value of 0.21 noted at the highest Cd concentration in soil (818 mg kg^−1^ d.w., Table 2).

**Figure 4:**
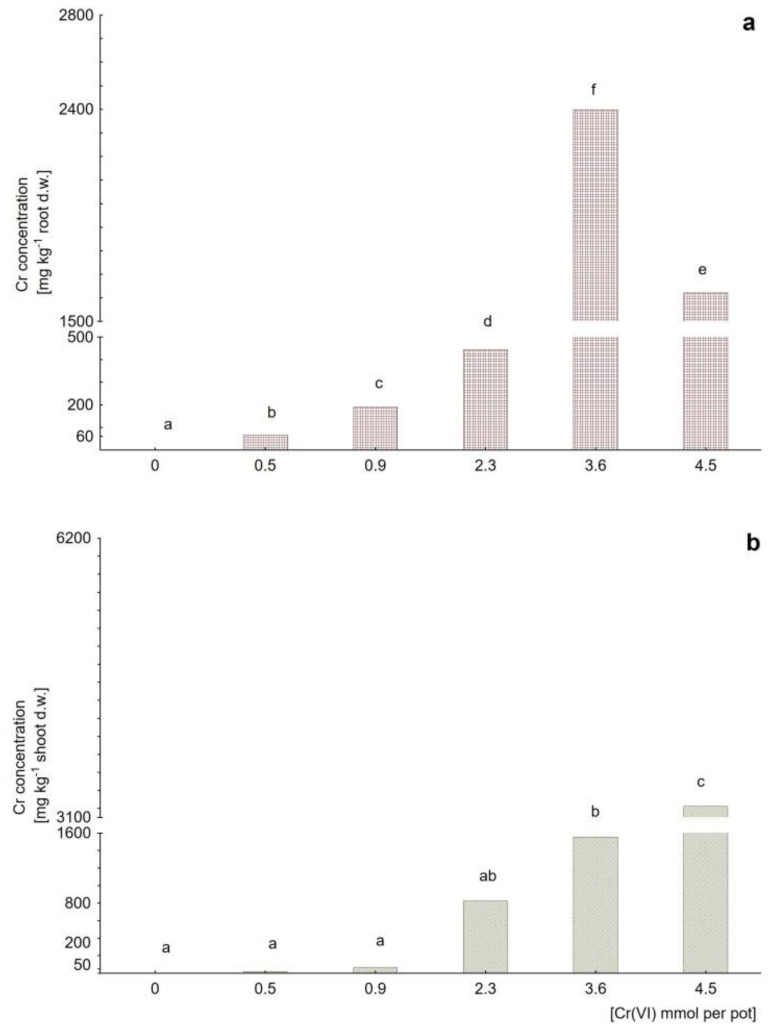
Chromium content in (**a**) roots and (**b**) shoots of *Mesembryanthemum crystallinum* plants subjected to Cr(VI) applied as potassium chromate, K_2_CrO_4_, at the doses of 0.5, 0.9, 2.3, 3.6 and 4.5 mmol per pot (untreated control at a dose 0). Different letters above the bars indicate statistically significant differences at *p* ≤ 0.05.

**Figure 5:**
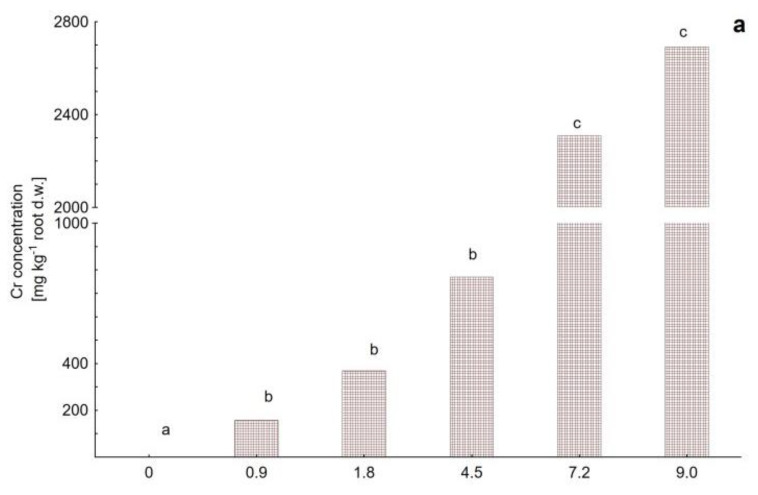

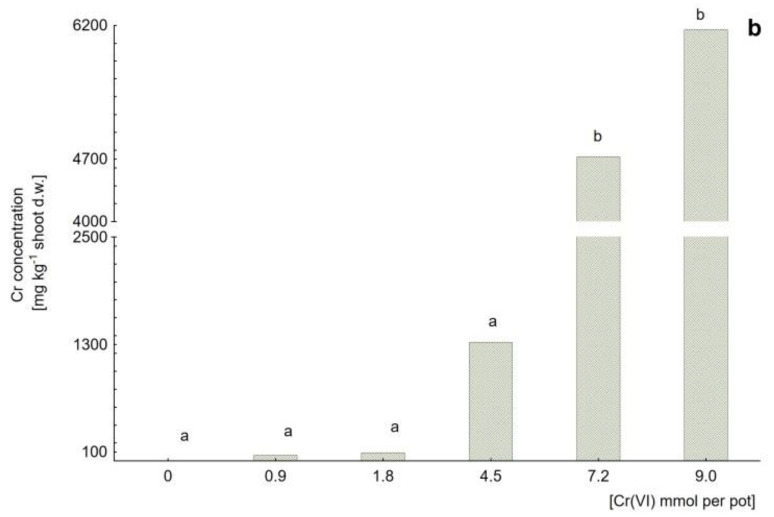
Chromium content in (**a**) roots and (**b**) shoots of *Mesembryanthemum crystallinum* plants subjected to Cr(VI) applied as potassium dichromate, K_2_Cr_2_O_7_, at the doses of 0.9, 4.5, 7.2 and 9.0 mmol per pot (untreated control at dose 0). Different letters above the bars indicate statistically significant differences at *p* ≤ 0.05.

*M. crystallinum* response to chromate treatment resulted in accumulation patterns different than that obtained for cadmium. For the case of potassium chromate model, the roots tended to accumulate Cr(VI) along with the rising concentration up to the dose determined as sublethal for the plant (that is 1699 mg kg^−1^ soil d.w.), at which the root-accumulated Cr reached a peak value of 2397 ± 1683 mg kg^−1^, Figure 4a). Then, at a dose of 2124 mg kg^−1^ the root-Cr level dropped to 1619 ± 437 mg kg^−1^. In accordance with the above, for lower chromate doses (that is at 236, 425, and 1086 mg kg^−1^ soil d.w.) it can be clearly seen that the shoots were protected against Cr(VI) entry, and the highest accumulated Cr level in aerial parts was determined as 823 ± 557 mg kg^−1^ (Figure 4b). Note however, that at sublethal and lethal doses (1699 and 2124 mg kg^−1^ soil d.w.), a significant increase in the shoot-accumulated Cr level was detected (2072 ± 1199 and 3221 ± 871 mg kg^−1^, respectively). Analogous reaction was observed for the dichromate model (Figure 5), where at Cr(VI) treatment doses of 2124, 3398 and 4248 mg kg^−1^ soil d.w. the shoots showed a tendency to increase Cr accumulation up to the highest value of 6152 ± 1211 mg kg^−1^ (Figure 5b), whereas in roots the Cr content was kept relatively low (maximum value of 2692 ± 538 mg kg^−1^, Figure 5a).

**Figure 6:**
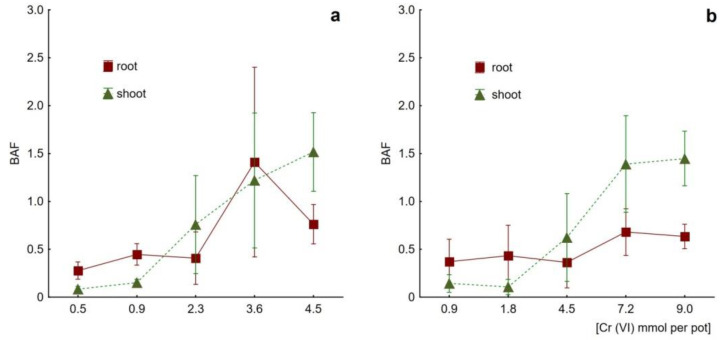
Chromium bioaccumulation factors (BAF) of *Mesembryanthemum crystallinum* roots and shoots as determined for plants subjected to treatment with chromate; (**a**) Cr(VI) applied as K_2_CrO_4_ at doses 0.5, 0.9, 2.3, 3.6 and 4.5 mmol per pot; (**b**) Cr(VI) applied as K_2_Cr_2_O_7_ at doses 0.9, 1.8, 4.5, 7.2 and 9 mmol per pot (N = 3, mean values ±SD).

For the common ice plant cultivated in the presence of chromate, the BAF parameter exceeded the hyperaccumulation threshold value of 1.0 at the doses of 3.6 and 4.5 mmol per pot (Figure 6a; K_2_CrO_4_ administration: BAF = 1.22 ± 0.71 and 1.52 ± 0.41 for doses of 1699 and 2124 mg Cr(VI) kg^−1^ soil d.w., respectively), and at 7.2 and 9.0 mmol per pot (Figure 6b; K_2_Cr_2_O_7_ administration: BAF = 1.39 ± 0.50 and 1.45 ± 0.29 for 3398 and 4248 mg Cr(VI) kg^−1^ soil d.w., respectively). Considering root-to-shoot Cr(VI) translocation, the TF values (Table 2) were kept relatively low (<0.4) for treatments up to 850 mg kg^−1^ soil d.w. (both Cr(VI) application models) and then climbed dramatically above 1.0 (TF = 1.72 for 2124 mg kg^−1^ soil d.w.), supporting the hyperaccumulation mechanism. The TF value peaked at 2.29 for the treatment dose of 4248 mg Cr(VI) kg^−1^ soil d.w.

## 3. Discussion

Environmental reclamation biotechnologies are focused on reimposing original, optimal soil-water characteristics to recover biological balance and bring back opportunities for future sustainable exploitation. A number of plant species have been shown to be potentially applicable in terms of promoting successful revitalization of degraded areas by variant processes. Such plants are expected to resist harsh environmental conditions and tolerate polluting agents, and in many cases of soil remedial projects they should reveal adaptive mechanisms to high salinity and contamination with heavy metals and/or xenobiotics.

The common ice plant (*Mesembryanthemum crystallinum*) has long been recognized as a suitable model for studying plant adaptive mechanisms to extreme conditions [47]. As a semi-halophyte, it was shown to be able to carry out complete life cycle under NaCl concentrations reaching 800 mmol L^−1^ [41]. Moreover, due to its original desert habitats (the Namib Desert in southern Africa), the plant has evolved enhanced tolerance towards variety of physiological stresses, enabling it to grow in conditions of low nutrient content, water deficit, poor soil structure, and highly variant diurnal temperatures. For the above reasons *M. crystallinum* can be proposed as an efficient plant in phytoremediation of heavy metals and reclamation of saline soils. It is important to notice that halophytic plants have been suggested as favorable candidates for use in heavy metal remedial projects; first, because of the frequent concurrence of both salt and heavy metals as polluting agents [3,5,6] and second, because of common aspects of physiological response mechanisms to both stresses [5,50]. The ice plant has earlier been shown to accumulate Ni, Cu, Zn and Cd ions [3,46,47], although the evidence was only based on hydroponic cultures.

In this study, an attempt was made to reveal the plant response to treatment with high levels of Cd(II) and Cr(VI) in soil pot tests, that is under conditions resembling real environmental sites. Tóth et al. [51] gives the threshold and guideline values for various metals and metalloids in soils. The lower and higher guideline values were based on the ecological or health risk (for Cd the values range from 10–20 mg kg^−1^ and for Cr, 200–300 mg kg^−1^). According to the survey on European soils carried out by these authors the excessive levels of Cd are regional-specific whereas Cr is abundant in most topsoils and 1.1% of the tested samples are above the guideline value, proving that approximately 2 million ha of agricultural land are at an ecological risk and require remediation actions. Taken the extreme tolerance of *M. crystallinum* to both Cd and chromate ions we propose that this plant, apart from reclamation of topsoils, might be used for efficient phytoremediation in revitalization projects of heavily polluted areas such as dumps of slurries and sediments (e.g., that of the tannery industry), post-mining, post-flotation wastes, fly ashes as well as other industrial waste heaps, where both the Cd and Cr concentrations might be many-fold greater than the guideline value ranges [51].

For cadmium treatment, our observations support the recently published data proving high tolerance towards this metal [48,49], for plants performing both the C_3_ and CAM photosynthesis. It should be noted, however, that possibly a significant fraction of Cd supplemented to the soil substrate was biologically unavailable for the plant since it is well known that Cd can get involved in various interactions with the soil leading to its gradual fixation and immobilization [52]. Both the kinetics of this process and the final Cd bioavailability depend on several physical-chemical factors and soil properties (pH, redox potential, presence of carbonates, chelators, other metals, activity of soil microbiota), as well as on the agricultural land use and afforestation [16]. The abovementioned phenomena might decrease the toxicity of the bulk cadmium added to the soil as only the mobile forms of Cd^2+^ can be taken up and then interfere with cellular structures and processes. In this study, the quantitative analysis of the ratio of extractable Cd portion (extraction with 1 mol L^−1^ HCl) to the total amount of the metal administered as CdCl_2_, yielded the average value of 35% ± 14% (for all the tested doses, data not shown). Accordingly, for the highest Cd dose applied (the total of 818 mg kg^−1^ soil d.w.) and considered sub-lethal (see Figure 1b), the Cd concentration calculated as potentially biologically available reached 244.4 mg kg^−1^ soil d.w. Note that such a level is still very high when compared to the available published information on cadmium toxicity. In a recent thorough review of the cadmium data collected to date, He et al. [12] describe variety of physiological and biochemical strategies developed by plants to cope with the Cd toxicity and critically discuss the applicability of both hyperaccumulating and non-hyperaccumulating plant ecotypes in phytoremediation of Cd contamination. The authors conclude that most plants reveal visible toxicity symptoms at a total soil Cd level of 8 mg kg^−1^. At the same time, bioavailable portion of Cd becomes toxic at concentrations as low as 0.001 mg kg^−1^. For tissue-accumulated cadmium, detrimental effects were observed for the levels ranging from 3.0 to 30 mg kg^−1^_,_ whereas the highest Cd concentrations applied were 160 mg kg^−1^ soil d.w. Another review of Rizwan et al. [14], dealing with the Cd-tolerance in vegetables, brings data on the soil pot experiments in which toxicity effects (interference with the nutrient uptake) were generated upon cadmium administration of 1–5 mg kg^−1^ soil. The highest tested level of 60 mg Cd kg^−1^ soil was described for cultivated potato, where in a long-term (60-day) treatment significant growth inhibition of seedlings was reported together with decreased chlorophyll and carotenoid contents as well as impaired nutrient uptake [53]. In the light of the above, *M. crystallinum* indeed proves to have evolved a cadmium-resistant phenotype. It has to be recognized, however, that the cited data represent different experimental models of Cd treatments, as described in particular papers.

In earlier studies, *M. crystallinum* was only tested for cadmium tolerance in hydroponic [46] and perlite-based [54] cultures, in which Cd was supplied directly, in an easily available metal salt soluble form. Decreased plant biomass yield was shown at 10 μmol L^−1^ Cd [46] and strong growth inhibition accompanied by decreased chlorophyll concentration was observed at 50 μmol L^−1^ Cd [54]. These toxic concentrations were comparable with the ones described above for different plants [12,14] and, unlike our soil test results, did not indicate any special resistance mechanisms towards cadmium.

To our knowledge, there is no available data regarding the effect of chromium on *M. crystallinum*. This is thus the first report showing the plant response to this heavy metal, applied as a chromate anion. The observed common ice plant extraordinary tolerance to Cr(VI) can only be compared with some other terrestrial plant models examined for the chromium effect. Importantly, different treatment conditions and experimental setups have to be considered, which make the obtained data difficult to interpret. In the review of Zayed and Terry [18], total soil Cr concentrations of the range 75–100 mg kg^−1^ were described as threshold toxicity levels for most plants. For the case of *M. crystallinum* no phytotoxicity symptoms were observed for Cr(VI) doses below 1699 mg kg^−1^ soil. However, it has to be pointed out that chromate was administered gradually (upon 9-day treatment), possibly allowing the plant to adapt to the toxic action and to induce resistance mechanisms. Chromate tolerance is in fact determined by multiple factors (see Prado et al. [23] for discussion) such as the applied treatment conditions and soil characteristics. All these circumstances should be considered since they determine generation of specific Cr forms acting directly on roots. Then, variety of Cr biotransformation reactions occur upon uptake, especially Cr(VI)→Cr(III) reduction carried out within plant tissues [25,55,56]. It is known that toxicity effects result mainly from the action of the soluble Cr species, easily accessible to plant cells [21,25]. In soils rich in humic substances and organic matter, large portions of Cr may become immobilized due to such processes as metal adsorption, reduction, and/or precipitation [23]. In consequence, only a small fraction of Cr is usually bioavailable to plants under conditions of soil cultivation [16,18,23]. It is therefore expected that in our case the peat-based substrate used in the experiment had a relatively high chromate reducing and adsorbing capacity, reflected by high ratio of the Cr immobilized fraction. In order to determine the amount of Cr potentially bioavailable to plants, a 1 mol L^−1^ HCl soil extraction yielded average values of 3.8% ± 1.6% and 3.1% ± 0.4% of the soil-extractable chromium, for treatments with potassium chromate and potassium dichromate, respectively. It was then calculated that for the two Cr(VI) sources, the sublethal doses of 1700 and 3400 mg kg^−1^ contained approx. 65 and 105 mg kg^−1^ of mobile Cr fractions, respectively. The latter values can now be compared with the results of other authors [18] who showed that for the plant-accessible Cr form, concentrations as low as 1–5 mg kg^−1^ were typically shown to be severely toxic. Thus, the data of our study imply that the ice plant retained undisturbed morphology upon treatment with relatively very high chromate doses and suggest that that *M. crystallinum* reveals extraordinary adaptive properties towards chromium.

Taken together, the collected data prove high tolerance of the common ice plant to both cadmium and chromate presence. At this stage it is very difficult to explain plant reactions to each of the studied heavy metals in terms of possible induced resistance mechanisms since the physiological response is complex and depends on a variety of environmental factors. Therefore, a more detailed study is required to account for the observed facts. In particular, it is of interest to verify whether *M. crystallinum* has an enhanced potential to cope with the oxidative stress generated by heavy metal treatment as this plant is known to reveal high antioxidant potential and to produce high activities of antioxidative enzymes when subjected to physiological stressors [42]. In order to shed some light on the mechanisms of resistance towards Cd and chromate, oxidative stress parameters should be determined as well as antioxidative enzymes and non-enzymatic antioxidants. Some data are already available with regard to superoxide dismutase (SOD) isoforms upon Cd treatment of *M. crystalinum* [48]. They suggest very complex processes involved in the reaction to the heavy metal stress. The cytoplasmic CuZnSOD activity was not elevated by Cd treatment, while Cd tended to induce the SOD activity in a compartment-dependent manner. However, thorough analysis of the common ice plant oxidative stress response requires a separate study based on systematic experimental setup. This is because the antioxidative status of *M. crystallinum* is a multifaceted problem and depends on several different factors. The plant may react to abiotic stress by switching its photosynthetic metabolism from C3 to CAM, which involves profound changes in the antioxidative system. Heavy metal stress might induce some metabolic changes typical of CAM and thus affect activities of several enzymes including the antioxidant ones. In addition to that, we earlier showed that important enzymes participating in the oxidative stress response such as catalase and SOD fluctuated according to daily rhythms [45,57,58] and, moreover, the activities of SOD isoforms were modulated upon abiotic stress in a compartment-specific manner [45]. These fluctuations could interfere with the response specific to the heavy metal presence. Also, the consortial structure and population dynamics of rhizospheral microbiota should be examined upon treatments since microbial activity has been suggested as critical for transformations of heavy metals in soil, their availability, toxicity and uptake by plants [7,14,23].

The Cd accumulation data indicate that the plant tended to retain cadmium within roots, not allowing for intensive metal translocation into the photosynthetic part. In consequence, hyperaccumulation in leaves can be excluded taken the proposed criterion of 100 mg kg^−1^ [7,59]. The BAF parameter calculated for shoots was initially low (≤0.52) and decreased further along with increasing Cd concentrations. In shoots, BAF also tended to decrease at higher Cd doses (cf. Figure 3). These results clearly suggest that the ice plant can induce some avoidance mechanisms in order to protect its aerial parts against the entry of toxic cadmium cations. Also, the low value of TF (TF = 0.21, Table 2) should be interpreted as typical of the excluder plants [23].

The plants revealing metal-accumulation characteristics as described above can only be used to stabilize soils, provided they can tolerate high metal concentrations. Consequently, based on the data of *M. crystallinum* reaction to Cd treatment, it is suggested that the common ice plant may prove efficient in phytostabilization of areas heavily polluted with cadmium and may prevent the Cd contamination from further migration into ground or water environments.

For Cr(VI) treatment, two models were applied, which were based on irrigation with either potassium chromate or potassium dichromate solutions to provide the plants with chromate ions. Although it is very difficult to trace the transformation paths of chromate within the soil environment (see Prado et al. [23] and Kabata-Pendias [16], for discussion on Cr behavior and bioavailability in soils), it was found that for both models the fraction of HCl-extractable chromium was similar (3.8% and 3.1%, respectively).

Toxicity threshold levels were different for each treatment model (1699 and 3400 mg Cr calculated as a chromate ion per kg of soil d.w., respectively) enabling the dichromate model to test higher administered Cr(VI) doses. Despite the observed toxicities at the elevated chromate levels, in short-term experiments it was of interest to examine even the highest Cr(VI) doses (see Table 2) since the morphological injuries might result from Cr accumulation and possible translocation to leaves. Such an approach was found particularly interesting because it could be expected that at extreme Cr concentrations the stressed plants might tend to reveal accumulation capabilities enabling efficient Cr removal from the soil. This, in turn, would further confirm the enhanced *M. crystallinum* phytoremediation potential towards this heavy metal.

The obtained Cr-accumulation patterns, especially resulting in accumulation of the metal in shoots at extreme Cr(VI) doses may be explained by the plant capability of launching some root-to-shoot translocation mechanism under chromate stress, which led to elevated levels of Cr in leaves and possibly caused toxicity. This is unusual since most plants studied so far tended to retain Cr upon its entry inside the root thus avoiding translocation into shoots [23,60]. Such strategy is justified by the fact that the toxicity effects correlated with the amount of Cr translocated into shoots and accumulated in leaves [18,23]. In many cases Cr(VI) concentrations in leaves ranging from 1–10 mg kg^−1^ caused toxicity manifestations [18], while accumulation of >65 mg kg^−1^ led to very strong adverse effects. The levels of >100 mg kg^−1^ were found typically lethal [60], and only in a few cases [16,18] the resistant plants could accumulate over 100 mg kg^−1^. At the same time, the common ice plant, when treated with potassium chromate, developed first toxicity symptoms at the shoot accumulation level of 2072 ± 1199 mg kg^−1^ (cf. Figure 1c and Figure 4b) upon soil-administered dose of 1699 mg kg^−1^. No inhibitory effects were observed for the value as high as 823.8 ± 557 mg kg^−1^ of accumulated Cr (that is, at applied dose of 1086 mg kg^−1^ soil d.w.), which makes the common ice plant hypertolerant to chromate. Preliminary data on the photosynthesis apparatus of *M. crystallinum* treated with Cr(VI) showed stability of photosystem II photochemical parameters up to 253 mg Cr(VI) kg^−1^ soil, which further confirmed high resistance towards chromate (unpublished data). It was postulated that *M. crystallinum* achieved its high tolerance to environmental stress by evolving specific adaptive physiological and biochemical mechanisms consisting in accumulation of acids (especially malate) inside leaf vacuoles [61]. It is thus tempting to suggest that this phenomenon, based on the activity of anionic carriers and the V-type H^+^-ATPase could add to the plant’s exceptional resistance to chromate and to its tendency to accumulate Cr compounds in aerial parts at high Cr(VI) concentrations. However, further studies are required to establish the detailed mechanisms of chromium uptake, translocation to shoots, possible bioreduction, and final distribution of the sequestered Cr within the target cell organelles.

High levels of Cr determined in *M. crystallinum* shoots indicate the presence of an efficient accumulation system. According to the proposed criteria for chromium (300 mg kg^−1^) [59], a hyperaccumulation mechanism can be suggested, although such a capability should be confirmed under field conditions as emphasized by van der Ent et al. [59]. As pointed out by other authors [23,59,60] there are very few species capable of Cr hyperaccumulation. Prado et al. [23] describe 13 species evidenced in a recent literature (dated 2010 or later) as being able to hyperaccumulate Cr, assuming the 1000 mg kg^−1^ threshold. In the present study we emphasize that the peak value of Cr accumulation by the ice plant (6152 ± 1211 mg kg^−1^, Figure 5b) indicates indeed very high accumulation capacity, comparable with the best chromate hyperaccumulators reported ever. Shahandeh and Hossner [60] documented ragweed and vetiver grass accumulation of 7000 and 10,000 mg kg^−1^ at 500 mg kg^−1^ of Cr(VI) administered to the soil (lethal doses). The highest to date reported Cr hyperaccumulation potential was assessed by Kalve et al. [62] for *Pteris vittata*, capable of phytoextracting As and Cr, and accumulating Cr up to 20,675 mg kg^−1^.

The analysis of BAF and TF parameters supports the idea of the induced hyperaccumulation of chromate resulting from its translocation from roots to shoots under treatment with high Cr(VI) doses. In general, it is assumed that plants characterized by both the BAF_shoot_ and TF_root-to-shoot_ values greater than 1.0 are capable of hyperaccumulation and efficient phytoextraction and are thus applicable to remove heavy metals from soil upon harvest of the aerial parts [7,23,59,60,63]. Both criteria are met by the values obtained for shoots of *M. crystallinum* treated with the higher Cr(VI) doses (cf. Figure 6 and Table 2). It is noteworthy that the calculated BAF parameters may underestimate the actual Cr accumulation capability since these factors were obtained assuming the total amount of Cr added (bioavailable + immobilized fractions). Taking only the extractable fraction for calculations, as determined with the ICP-OES technique, the resultant BAF_shoot_ parameters are about 20–30 times higher (data not shown). Finally, considering all the Cr(VI) accumulation data, *M. crystallinum* proves to fulfill conditions required for its suggested use in Cr phytoremediation projects.

## 4. Materials and Methods

### 4.1. Plant Cultivation and Treatment with Heavy Metals

Common ice plants (*Mesembryanthemum crystallinum* L.) were grown in 0.5 L pots (100 × 75 × 75 mm) on the substrate obtained upon mixing of the market-available universal soil substrate “Hartmann” (Hartmann Polska, Poznań, Poland) with sand (proportions: 7.5 L substrate: 1 kg sand). The “Hartmann” soil contained milled high peat, fraction 0–20 mm, pH 5.5–6.5, supplemented (1.0–1.3 kg m^−3^) with the all-in-one powdered multicomponent fertilizer “PG Mix NPK 14:16:18”. The final elemental content of the substrate was determined with the method of ICP-OES (see below) as biologically available macro- and micro- nutrients upon soil substrate extraction with acetic acid. The concentrations of P, K, Mg, Ca, S, Cu, Fe, Mn, Al, Ba, Li, Na, and Sr were: 29.89, 60.69, 61.71, 1062.9, 14.93, 0.11, 0.24, 0.37, 0.76, 0.31, 0.01, 25.69, 0.68 mg per L, respectively. Nitrogen was present as nitrites (III) and nitrates (V) (a total of 13 mg L^−1^) and ammonia (53 mg L^−1^). The final substrate dry weight (d.w.) applied per pot was determined as 0.11 kg. Soil substrate acidity (pH of 5.5–5.7) and overall salinity (EC of 1–1.5 mS cm^−1^) were measured with potentiometric and conductometric techniques, respectively, in a soil: water mixture (20:40 cm^3^).

*M. crystallinum* seeds were sown and the sprouts grown for three weeks. Then, prior to the heavy metal treatment procedures, the seedlings were quilted to individual pots, where they continued to grow for 20 days. The experiment concerning plant resistance and heavy metal accumulation was carried out in a summer season (July/August 2018), in a greenhouse under the temperature ranging typically from 20 °C (morning hours) to +30 °C (early and late afternoons) and reaching occasionally + 40 °C on some warm and sunny days (the latter was observed as not to have any negative effect on the plant growth). The plants were irrigated each day with the appropriate heavy metal solutions or with water (control). The treatment conditions covered a wide range of concentrations, so as to apply both the non-inhibitory and non-toxic heavy metal doses as well as doses that led to toxicity symptoms. The tolerance against the tested heavy metals was established based on determination of physiological and morphological characteristics such as growth inhibition, necrosis and chlorosis.

For the case of cadmium, 10 mL volumes of 0.01, 0.1, 1.0, and 10 mmol L^−1^ CdCl_2_ (Sigma-Aldrich, Poznań, Poland) were applied daily for eight days. Such treatment led to the final Cd doses of 0 (control), 0.8, 8.0, 80 and 800 μmol per pot, respectively, which were calculated as 0.82, 8.2, 82 and 818 mg per kg of soil d.w. For chromium, the plants were treated employing two experimental models, in which either potassium chromate (K_2_CrO_4_) or potassium dichromate (K_2_Cr_2_O_7_) (both Sigma Aldrich) were used. These variant approaches were necessary because the plants responded differently towards treatments with each chromate source. Both Cr(VI) salts were applied at initial concentrations of the irrigation solution of 5, 10, 25, 40 and 50 mmol L^−1^. The daily irrigation of 10 mL aliquots was carried out for nine days. It was then calculated that, for the case of K_2_CrO_4_ and K_2_Cr_2_O_7_, the resultant Cr(VI) final doses per pot were equal to: 0.5, 0.9, 2.3, 3.6, 4.5 mmol (that is 236, 425, 1086, 1699, 2124 mg kg^−1^ soil d.w.), and 0.9, 1.83, 4.5, 7.2, 9.0 mmol (that is 425, 850, 2124, 3398, and 4248 mg kg^−1^ soil d.w.), respectively.

### 4.2. Biometric Analyses and Determination of Heavy Metals in Plant and Soil Samples

Having completed the experiments, the whole plants were collected and the remaining soil substrate stored at 4 °C. The plant material was divided into the root and shoot parts. For biometric analyses the collected root parts were rinsed with cold distilled water until soil remnants were removed; the shoots were also rinsed briefly and together with roots blotted with filter papers. The fresh weight was measured immediately, whereas dry weight was determined upon 48 h desiccation in an oven at 105 °C.

For determination of the heavy metal content in *M. crystallinum* roots and shoots, the plant tissue samples were mineralized in 65% HNO_3_. The method of ICP-OES (inductively coupled plasma-optical emission spectrometry) was employed (Prodigy Teledyne Leeman Labs, Mason, Ohio, USA), based on appropriate calibration curves with the Certipur^®^ reference standards (Merck, Darmstadt, Germany), namely the ICP multielemental standard IV (1000 mg L^−1^ solutions of 23 elements in dilute nitric acid): Ag, Al, B, Ba, Bi, Ca, Cd, Co, Cr, Cu, Fe, Ga, In, K, Li, Mg, Mn, Na, Ni, Pb, Sr, Tl, Zn”.

The analyses of cadmium and chromium concentration in soil samples were done according to the method of Rinkis, based on the extraction of 10 g specimens with 1 mol L^−1^ HCl, as earlier described [64,65]. Then, the extracts were subjected to ICP-OES as described above. Note that the described analytical approach allowed for determination of more than just a soluble fraction of a given metal, enabling to extract also ionic metal fractions that were exchangeable and weakly adsorbed to the substrate particles. It was assumed that the extracted material represented the portion of the tested metal which was biologically available to plants.

### 4.3. Evaluation of Heavy Metal Accumulation Capabilities: Bioaccumulation and Translocation Factors

In order to characterize strategies employed by the common ice plant towards Cd and Cr presence, bioaccumulation factor (BAF) and translocation factor (TF) were determined for all the administered metal doses. BAF was defined as heavy metal concentration [mg kg^−1^ d.w.] in plant organs (roots or shoots) per total concentration in the soil substrate [mg kg^−1^ soil d.w.]. TF was calculated as the ratio of a heavy metal accumulated in shoots to that determined in roots [mg kg^−1^ d.w.].

### 4.4. Statistical Data Analysis

All the analyses were done in triplicate. The results were statistically evaluated with the one-way ANOVA module of the Statistica 13.3 software (StatSoft Polska, Kraków, Poland), employing a Duncan’s post-hoc test at the significance threshold level *p* ≤ 0.05.

## 5. Concluding Remarks: Evaluating *M. crystallinum* Biotechnological Applicability

*Mesembryanthemum crystallinum* can inhabit sites characterized by harsh conditions including dry, saline and polluted soils. It grows relatively fast (its vegetation cycle lasts for about 6 weeks) while producing well-developed root system. Within the root zone favorable conditions are provided for proliferation of rhizospheral microbiota which can contribute to plant tolerance towards physiological stress agents. The present study brings evidence on very high tolerance of the common ice plant to heavy metals as exemplified by cadmium cation and chromate anion.

In soil pot tests the heavy metal-treated plants revealed different strategies of coping with the action of toxic agents. For Cd^2+^ administration, an excluder’s activity prevented both roots and shoots from excessive metal accumulation. For the case of chromate, enhanced accumulation potential was documented with a tendency to translocate Cr(VI) into shoots at high (sub-lethal to lethal) treatment levels. All the described capabilities make *M. crystallinum* a good choice to cultivate as a pioneer plant in efforts to biologically reclaim anthropogenically degraded soils. For cases of pollution with cadmium the common ice plant could be applied to phytostabilize polluted sites, enabling further colonization with beneficial microbiota, which might modify the soil structure and heavy metal availability to make such an area more susceptible for further remediation actions. For chromate contamination, the mechanism of stress-induced translocation of chromium from roots to shoots resulting in shoot hyperaccumulation suggests that the plant can be used for phytoextraction of Cr contamination.

## Figures and Tables

**Table 1:** Weight of whole plants, roots and shoots determined in a soil pot test upon treatment of the common ice plant, *Mesembryanthemum crystallinum* with Cr(VI) administered as potassium chromate or potassium dichromate.

Treatment	Cr(VI) Dose [mmol per pot]	Plants f.w. [g]	Plants d.w. [g]	Roots d.w. [g/plant]	Shoots d.w. [g/plant]
Cr(VI) (K_2_CrO_4_)					
	0	13.03 ± 1.12 a	1.16 ± 0.19 a	0.07 ± 0.00 a	1.09 ± 0.19 a
	0.45	15.30 ± 0.20 a	1.15 ± 0.06 a	0.06 ± 0.01 a	1.09 ± 0.05 a
	0.90	12.52 ± 2.62 a	0.99 ± 0.21 ab	0.06 ± 0.00 a	0.94 ± 0.21 a
	2.30	11.09 ± 2.96 b	1.05 ± 0.18 b	0.07 ± 0.02 a	0.99 ± 0.18 a
	3.60	7.75 ± 3.49 b	0.81 ± 0.39 b	0.06 ± 0.02 a	0.75 ± 0.37 a
	4.50	4.06 ± 0.78 c	0.91 ± 0.04 a	0.06 ± 0.01 a	0.85 ± 0.04 a
Cr(VI) (K_2_Cr_2_O_7_)					
	0	13.03 ± 1.12 ab	1.16 ± 0.19 a	0.07 ± 0.00 b	1.09 ± 0.19 a
	0.90	15.53 ± 1.28 b	1.16 ± 0.07 a	0.05 ± 0.02 a	1.11 ± 0.04 a
	1.80	12.42 ± 2.05 a	1.03 ± 0.11 ab	0.06 ± 0.01 ab	0.97 ± 0.11 ab
	4.50	13.43 ± 1.46 ab	1.18 ± 0.12 a	0.06 ± 0.01 ab	1.12 ± 0.12 a
	7.20	3.67 ± 0.26 c	0.88 ± 0.16 b	0.06 ± 0.00 ab	0.82 ± 0.16 b
	9.00	4.26 ± 1.71 c	1.01 ± 0.07 ab	0.05 ± 0.00 a	0.96 ± 0.08 ab

Cr(VI) doses were calculated as chromate ions; mean values within columns followed by the same letters are not significantly different at *p* < 0.05 according to Duncan’s test (N = 4, mean value ± SD).

**Table 2:** Cd and Cr translocation factors (TF) calculated upon treatment of the common ice plant, *Mesembryanthemum crystallinum*, in a soil pot test.

Treatment	Dose [mmol per pot]	Dose [mg kg^−1^ soil d.w.]	TF (shoot/root)
Cd^2+^ (CdCl_2_)			
	0.0008	0.82	0.33
	0.008	8.20	0.33
	0.080	82.0	1.00
	0.800	818	0.21
Cr(VI) (K_2_CrO_4_)			
	0.45	236	0.31
	0.90	425	0.34
	2.30	1086	1.86
	3.60	1699	0.86
	4.50	2124	1.99
Cr(VI) (K_2_Cr_2_O_7_)			
	0.90	425	0.39
	1.80	850	0.25
	4.50	2124	1.72
	7.20	3398	2.05
	9.00	4248	2.29

All the Cr(VI) doses were calculated as chromate ions; the doses expressed in [mg kg^−1^ soil d.w.] were recalculated based on the applied molar concentrations; TF was calculated as the ratio of a heavy metal accumulated in shoots to roots [mg kg^−1^].
